# Occupational Self-Efficacy and Psychological Capital Amongst Nursing Students: A Cross Sectional Study Understanding the Malleable Attributes for Success

**DOI:** 10.3390/ejihpe10010014

**Published:** 2019-10-26

**Authors:** Daniel Terry, Blake Peck, Andrew Smith, Hoang Nguyen

**Affiliations:** 1 School of Nursing and Healthcare professions, Federation University Australia, Ballarat 3350, Australia; b.peck@federation.edu.au (B.P.); andrew.smith@federation.edu.au (A.S.); 2 Wicking Dementia Research and Education Centre, University of Tasmania, Hobart 7001, Australia; hoang.nguyen@utas.edu.au

**Keywords:** nurses, nursing, student, psychological capital, self-efficacy, training, experience, workforce

## Abstract

With a predicted shortfall in the worldwide nursing workforce, efforts to understand attributes that influence attrition and workforce longevity remain fundamental. Self-efficacy and the broader construct of psychological capital have been linked to positive workplace-based attributes in occupations. The aim of the study was to examine the relationship between general self-efficacy, occupational (nursing) self-efficacy, and psychological capital and their predictive factors among nursing students. A cross sectional design was used to address the aims of the study where all nursing students studying a three-year bachelor’s degree were invited to complete a questionnaire examining traits that might assist in the preparation for, and longevity in, a nursing career. Although the participating nursing students demonstrated high levels of general self-efficacy, their reported levels of nursing-specific self-efficacy were significantly lower. Psychological capital measures indicated that students had high levels of belief, hope, and resilience concerning their capacity to commit to and achieve goals, succeed now and into the future, and overcome obstacles. The findings suggest an opportunity exists for education providers to nurture the malleable aspects of self-efficacy and psychological capital, while developing greater capacity to bounce back and overcome the challenges that nursing students may encounter in their undergraduate academic training, and to reduce attrition as they prepare to enter the workplace.

## 1. Introduction

Global populations are rapidly aging, increasing the demand for nurses, but a shortfall of nurses is projected [1]. This shortage is made worse with many new nurses leaving the profession within five years of graduation [1]. Nurses tend to leave due to workload, workplace culture, and emotional strain, signaling a need for better insights into how we might more adequately prepare students for the rigors of the profession [2]. One approach is developing greater self-efficacy and the broader construct of psychological capital, which have been linked with positive workplace-based attributes in occupations [3,4,5].

Among higher education students, lower levels of occupational self-efficacy and psychological capital have been shown to lead to lower academic outcomes with higher dropout rates [3,4,5]. Likewise, in the workplace, lower levels of self-efficacy and psychological capital leads to poorer work outcomes with lower levels of job satisfaction, greater job stress, and poor coping skills [3,4,5]. The inverse is also explanatory amongst first-year nursing students with higher occupational self-efficacy which is predictive of third-year final marks for the same students and correlated positively with lower attrition and higher program completion rates [6]. 

In general, it has been shown that occupational self-efficacy and psychological capital constructs are malleable and open to change in the development of self [7]. An individual who develops the major sources of efficacy—personal mastery, experiences of others, verbal persuasion, and emotional arousal, has the propensity to improve self-efficacy, which leads to improved academic and occupational outcomes [8]. Further, as outlined by Luthans, et al. [4], building optimism, resilience, and hope to enhance psychological capital can lead to greater cognitive and behavioral plasticity, social competence, problem solving skills, and autonomy [9]. However, it is argued that efforts to develop the core constructs of self-efficacy and psychological capital remain absent within the literature, are ineffectual, or not well implemented [7]. Thus, it is known that self-efficacy and psychological capital are vital to student academic outcomes and better workplace satisfaction; however, the factors that impact the development of these attributes, specifically among nursing students, remain elusive [3,4,5,6,7].

### 1.1. Context of Self-efficacy and Psychological Capital

Self-efficacy reflects an enthusiastic self-belief that an individual can perform novel or difficult tasks, or cope with challenges [10,11]. Self-efficacy beliefs are similarly defined by Bandura and Locke (2003) as “rooted in the core belief that one has the power to produce desired effects” [5] (p. 87). The concept of self-efficacy is in parallel with social cognitive theory, which emphasizes social influence, social reinforcement, and the ability to adapt to change [10,11]. For example, self-efficacy enables goal-setting, the investment in effort, and the persistence to face barriers and recover from setbacks or failures [8]. Overall, self-efficacy has been regarded as a positive factor of resistance, and one that can impact success among university students in regard to both academic outcomes and career choices [10,11].

In addition to the aforementioned, self-efficacy has been shown to determine job satisfaction, including performance in, and overall commitment to, a workplace [12]. Literature shows that general occupational self-efficacy scales have been used to understand individual commitment and work values, as well as the impact that manager behaviors have on influencing greater development of self-efficacy in the workplace [12]. Within the healthcare setting, similar self-efficacy scales have been used to identify employees with lower levels of self-efficacy who have a propensity for poorer coping skills, to burnout more rapidly, and who are less inclined to manage emotional distress well. 

Closely related, but broader than self-efficacy, is the construct of psychological capital, which is defined as “an individual’s motivational propensities and positive psychological state of development” [13] (p. 267). Psychological capital is a combination of attributes that include self-efficacy, hope, optimism, and resilience. Optimism is an individual’s belief of being successful combined with a positive, yet realistic, outlook concerning the capacity to succeed now and into the future. Hope refers to a positive motivational state and an individual’s ability to commit to goals, the willpower to achieve goals, and the capacity to create alternative pathways to overcome obstacles [14]. Lastly, resilience or endurance refers to the ability to withstand and bounce back or succeed after encountering problems or negative situations [13]. 

It has been shown that a positive relationship exists between psychological capital and academic, workplace performance and satisfaction among higher education students. The available research suggests that overall psychological capital scores are a better predictor of performance and satisfaction than the individual attributes alone [15]. Overall, the elements of self-efficacy, hope, resilience, and optimism reflect the capacity to successfully cope with challenges and imply an internal-stable acknowledgment of success [10,11].

### 1.2. The Current Challenge

There is very little literature that demonstrates the levels of self-efficacy and psychological capital among nursing students. In addition, not a lot of research has been done on the specific factors that have an impact on the development of self-efficacy and psychological capital among nursing students [6,16,17,18,19]. 

As such, the aims of this study were to:

1. Determine if general self-efficacy and nursing specific self-efficacy differs among nursing students in terms of age groups, regions (rural versus metropolitan), and previous learning backgrounds (with or without Enrolled nursing background);
2. Determine if there are correlations between general self-efficacy, nursing specific self-efficacy, and psychological capital among nursing students;
3. Determine the predictive factors of general and nursing self-efficacy among nursing students.

It was hypothesized that there is a positive association between general self-efficacy, nursing self-efficacy, and the four states of psychological capital. It is further hypothesized that self-efficacy and psychological capital differ among age groups, between rural and metropolitan students, and students with or without enrolled nursing backgrounds.

## 2. Materials and Methods

A cross-sectional design was used to examine traits among Bachelor of Nursing students that might assist in the preparation for, and longevity in, a nursing career. The study was conducted throughout an Australian university, which has campuses in rural, regional, and peri-urban centers.

### 2.1. Participants

All nursing students (n = 1982) studying the three-year Bachelor’s degree. 

### 2.2. Procedure

Students were invited to complete a suite of online questionnaires, between 28 June and 31 July 2018 [20]. The invitation to participate in the study included follow-up reminders in weeks 1, 2, and 4 post initial invitation. The invitation was sent by administration staff in the winter break to reduce impact on studies and inhibit coercion. No incentives were offered to participants. 

### 2.3. Data Measures

Data were collected using a questionnaire that included 23 demographic questions such as gender, year of birth, marital status, where the student grew up, and current employment. The questionnaire also included:

- the General Self-Efficacy Scale (GSE-10) developed by Schwarzer and Jerusalem [21] that contained 10 general self-efficacy items;
- the Nursing Self-Efficacy Scale (g-8) developed by Schyns and Von Collani [22] that had 8 occupation specific self-efficacy items. The scale was modified with minor wording changes, where the generic word ‘job’ was replaced with the specific word ‘nursing’; and
- the Psychological Capital Questionnaire (PCQ-12), which uses 12 items to measure four components that include (1) efficacy, (2) hope, (3) optimism and (4) resilience [4,23].

The GSE-10 and NSE-8 items were scored on a four-point scale with categories ranging from 1, Not at all true to 4, Exactly true. The total score for GSE-10 ranged between 10 and 40, and that of NSE-8 ranged from 8 to 32, with higher scores representing higher levels of self-efficacy in both scales [20,21]. PCQ-12 items were measured on a six-point scale with categories ranging from 1, Strongly disagree to 6, Strongly agree [23]. The total score for each sub-scale ranged between 4 and 24, with higher scores representing higher levels of psychological capital [23].

### 2.4. Ethical Considerations

Ethical approval was provided by the University’s Human Research Ethics Committee (A18-017).

### 2.5. Data Analysis

Data were cleaned, checked, and analyzed using Statistical Package for the Social Sciences (SPSS, Version 23.0). All responses for each of the three scales (GES, NSE, and PCQ-12) and four subscales within the PCQ-12 were summarized and averaged to get a mean score. Each of the scale and subscale mean scores were categorized into quartiles to give an insight into where students were located along the scale. 

Pearson’s correlation (r), independent sample t-test, one-way ANOVAs, and multiple linear regression were used to analyze data and identify differences according to age groups and students with or without enrolled nursing background, and regions. Preliminary analyses were undertaken to ensure no violations of assumptions were present. 

Correlation sample size required to adequately conclude whether a correlation coefficient differs from zero is: n = 196 (alpha [2 tailed] = 0.05, beta = 0.2, rho [expected correlation] = 0.2). Also, the sample size, n = 196, will have power to detect a 5% absolute difference within and between groups, (alpha (2 tailed) = 0.05, margin of error = ±5%. The strength of correlation was defined as large (r = 0.50–1.0), medium (r = 0.30–0.49), and small (r = 0.10–0.29). Significance was determined at two-tailed p ≤ 0.05; however, the p-value was adjusted using Bonferroni correction to account for the possibility of a Type I error rate resulting from multiple comparisons [24].

### 2.6. Validity and Reliability/Rigor

Cronbach Alpha (α) was used to test questionnaire item reliability within the current study and indicated that all questionnaires had acceptable reliability, GSE-10 = 0.858; NSE-8 = 0.749; and the PCQ-12 coefficients for Efficacy = 0.836, Hope = 0.863, Resilience = 0.649, and Optimism = 0.821 [24]. In addition, all questionnaires were demonstrated within previous studies to have good face and content validity [20,21,23], while the minor wording changes to the NSE-8 were also examined to have good face and content validity in the current study.

## 3. Results

The questionnaire was sent to all 1982 Bachelor of Nursing students, who were either first, second, or third year students undertaking the degree. Overall, 329 responded, yielding a response rate of 16.6%; however, 202 surveys (10.2%) were completed in full. Table 1 outlines the key demographics relevant to the aims of the study. The majority of students were female, mean age 32.54 years (SD 10.14), with a third of participants (32.5%) growing up in metropolitan areas, while one-fifth (21.1%) were currently working as enrolled (Division 2) nurses, and a sixth (15.5%) were unemployed.

**Figure 1:**
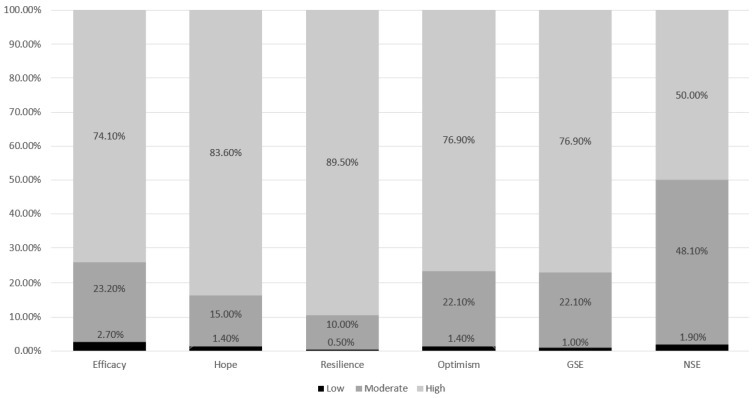
Psychological capital and general and nursing self-efficacy among nursing students.

The outcomes of the PCQ-12, GSE-10, and NSE-8 highlighted that among the cohort of nursing students, 85.0% had high levels of overall psychological capital scores (scores ≥ 24) (x̅ = 18.69, SD = 2.71) followed by 76.9% who had high levels (scores ≥ 31) of general self-efficacy (x̅ = 31.89, SD = 3.89), while 50.0% of students were shown to have a high level of nursing self-efficacy (scores ≥ 24) (x̅ = 23.91, SD = 3.01) as outlined in Figure 1. 

### 3.1. Differences between Groups

When examining the differences between GSE-10 and NSE-8, it was indicated that students had significantly higher levels of general self-efficacy than nursing self-efficacy, as outlined in Table 2. More specifically, general self-efficacy was shown to be significantly higher than nursing self-efficacy regardless of where students grew up, their employment status, or age group. The only exception was the 45 years and older cohort of students, as their general and nursing self-efficacy levels were not significantly different from one another (t(22) = 1.930, *p* = 0.67).

However, when examining general self-efficacy and nursing self-efficacy among the various groups of students, it was noted that there was no significant difference, except between students who grew up in metropolitan and rural areas. Metropolitan students had higher levels of general self-efficacy (32.85) and nursing self-efficacy (24.56) than their rural counterparts (31.32 and 23.15 respectively), while there were no significant differences between regional students and rural or metropolitan students. 

When examining individual psychological capital scale items against demographic factors, it was noted that students under 25 years of age had significantly lower levels of efficacy (4.11) than students aged 25 to 45 (4.59), while students from a metropolitan background had significantly higher levels of optimism (5.04) compared to both rural (4.66) and regional (4.53) students. Further, it was also highlighted that students who were unemployed had higher levels of hope (4.70) and resilience (4.85) than those students who were employed (4.49 and 4.49 respectively) as outlined in Table 2. It must be noted that there were no significant differences between males and females; however, this may be due to the low number of males in the cohort. 

Upon further examination, significant differences were found between students of different year levels. In all cases, first-year students had lower levels of efficacy and hope compared to third-year students, and lower levels of optimism than their second- and third-year counterparts. No significant differences between year levels in terms of efficacy were detected. This was also the case with GSE where there were no differences noted between year levels. However, first-year students were shown to have significantly lower levels of NSE than both the second- and third-year students, as outlined in Table 3. 

### 3.2. Multiple Regression to Establish Predictive Factors of General and Nursing Self-efficacy

Multiple regression analyses highlighted several significant predictors of students GSE and NSE. After controlling for age and gender, the combined effect of demographic factors (age, income) and several psychological capital items (Efficacy, Hope, and Optimism) explained 42.2% of the variance in general self-efficacy F(5, 168) = 24.575, *p* = 0.001. In addition, it was shown that Efficacy, Hope, income, and being an enrolled nurse were significant independent predictors of nursing self-efficacy, and explained 16.5% of the variance F(4, 182) = 12.682, *p* = 0.001, as outlined in Table 4.

### 3.3. Correlation between Self-efficacy Scales and Psychological Capital Scale Items

When examining the overall cohort for the association that exists between general and nursing self-efficacy, a medium positive correlation was identified. Further, when examining general and nursing self-efficacy, as well as the individual items and collective score of the psychological scale among students, it was noted there was a medium to strong positive correlation between general self-efficacy and all psychological capital items, as outlined in Table 5. In addition, there were small positive correlations between nursing self-efficacy and all psychological capital items, except resilience. 

When examining students in the various age groups for the association that exists between general and nursing self-efficacy, a large positive correlation was identified for those students aged under 25, and a medium correlation among those who were in the 25 to 44 and 45 and older age groups, as outlined in Table 6. However, these associations among the age groups showed a number of variations. For example, a medium to strong positive correlation occurred between general self-efficacy and psychological capital items, while a medium positive correlation between nursing self-efficacy and all psychological capital items occurred among the student under 25 years of age. However, it was noted that the older the age group, the smaller the correlation. For example, students 25 to 45 years of age had medium to large associations between general self-efficacy and psychological capital, with less correlation present for nursing self-efficacy. Conversely, those students aged 45 years of age and older had very few correlations between both general and nursing self-efficacy and psychological capital; however, the number of students in this group was relatively small. 

In terms of the association between general and nursing self-efficacy and individual items of the psychological capital scale among students from differing geographical locations, additional variations were noted. A medium positive correlation was apparent between general self-efficacy and most psychological capital items among student cohorts who grew up in metropolitan and regional areas, while a medium positive correlation was shown to occur between nursing self-efficacy and some psychological capital items among the metropolitan students only. Stronger correlations were evident among students who grew up in rural areas. For example, these students had larger associations between general self-efficacy, nursing self-efficacy, and psychological capital (Table 7). 

Additional variations were highlighted regarding the association between general and nursing self-efficacy, and each of the individual items including the collective score of the psychological scale among the students who were currently enrolled nurses, and those who were not. Overall, both large and small positive correlations were evident between general self-efficacy and psychological capital items among students who were enrolled nurses, while a medium positive correlation was shown to occur between nursing self-efficacy, optimism, and hope in the same cohort. Notably, larger correlations were found among students who were enrolled nurses than among those students who were not (Table 8). 

## 4. Discussion

The study highlights that although the nursing students demonstrated high general self-efficacy, their reported levels of nursing-specific self-efficacy were significantly lower, and there were significant differences between age groups, year level of study, where they grew up, and their employment status. This suggests that students, regardless of the factors outlined, tend to have higher levels of self-belief in terms of performing tasks or coping with challenges in general; however, students are likely to have much lower levels of self-belief concerning the performance of nursing tasks or coping with challenges that may arise in the nursing context, particularly among first-year students. Although not surprising, as nursing students are still learning the profession, it is noted that occupational self-efficacy has been shown to determine job satisfaction, including performance in, and overall commitment to, a workplace [3,4,5]. This suggests that the student cohort, although not yet in the workplace, may experience challenges in terms of satisfaction, commitment, coping, performance, and longevity once working as a registered nurse. 

Despite this foreseeable challenge, what was also demonstrated was the high level of psychological capital. This finding suggests that students, particularly second- or third-year students, have high levels of belief, hope, and resilience concerning their capacity to commit to and achieve goals, succeed now and into the future, and overcome obstacles by developing alternative pathways. Notwithstanding the low levels of nursing self-efficacy among students, specifically first year students, they demonstrated that through their psychological capital, they have the capacity to develop greater nursing self-efficacy and achieve greater levels of workplace performance and satisfaction [6,16]. 

Regarding differences between students of different regions, it was highlighted that metropolitan students had significantly higher levels of both general self-efficacy and nursing self-efficacy when compared to their rural counterparts. This finding suggests that metropolitan students may have had differing types of opportunities leading to the development of greater levels of self-efficacy, which may be related to differences in the day-to-day life of nurses when compared to their rural colleagues. However, with no differences between students who have grown up in regional areas and their rural and metropolitan counterparts, there may be a relationship between where students grew up in terms of the level of remoteness and the level of general self-efficacy and nursing self-efficacy. Specifically, it was found that the more rural, and thus further away from urban areas a student grew up, the more likely they were to exhibit lower levels of general self-efficacy and nursing self-efficacy. Similar findings were exhibited for optimism, whereby students who grew up in metropolitan areas reported higher levels of optimism than their rural and regional counterparts. These findings indicate where greater effort may be required to support those students from certain backgrounds, age groups, and employment status to develop general self-efficacy, nursing self-efficacy, efficacy, optimism, hope, and resilience. Despite this, other research among medical students had dissimilar findings [25,26]. 

Further, when examining general self-efficacy, it was shown to be influenced by the age of the students and their level of efficacy, hope, and optimism, as elements of psychological capital. As such, older students with higher levels of efficacy, hope, optimism, and lower levels of income demonstrated higher levels of general self-efficacy. Although unremarkable, this finding further confirms that life experiences, including challenges that come with age, may contribute to the development of greater levels of self-efficacy in general [8]. Also, nursing self-efficacy was found to be influenced by the student being an enrolled nurse, their level of hope and optimism, in addition to higher levels of income. Specifically, nursing students, who are also working as enrolled nurses with higher levels of hope, optimism, and income, had higher levels of nursing self-efficacy. In this sense, greater levels of nursing self-efficacy, the ability to believe and cope with the challenges, are to be found among those with previous nursing experiences where opportunities allowed them to develop higher levels of hope and optimism [7,8]. 

When examining the correlation between general self-efficacy and nursing self-efficacy, it was shown that the level of correlation between each factor was much higher in the younger age groups. In this case, it may be proposed that as younger nursing students’ levels of general self-efficacy and psychological capital increase, the level of nursing self-efficacy also increases. This was similar to where students grew up, where the strength of the correlation between general self-efficacy, nursing self-efficacy, and psychological capital were much higher among those students who grew up in rural areas, and declined or were not significant in the regional or metropolitan student groups. As rural nursing students’ levels of general self-efficacy and psychological capital increased, the level of nursing self-efficacy also increased, and although observed in the regional and metropolitan cohorts, the correlations were much smaller. Finally, this was also observed when examining correlation differences between students who were enrolled nurses than those who were not. It was demonstrated that correlations were, in most cases, much higher among students that were enrolled nurses than those who were not. 

Overall, the findings suggest that students who are younger, who are from more rural backgrounds, and who are enrolled nurses, are more likely to have higher levels of general self-efficacy and nursing self-efficacy if they have higher levels of psychological capital. This suggests there is a need to target younger people, and those who are rural and enrolled nurses to participate in baccalaureate nursing programs [6]. Despite this, the challenge is how to best address the current deficits observed to empower students and reduce both academic and professional attrition [4].

### 4.1. Developing Greater Self-efficacy among Nursing Students

In moving forward, nursing students have the capacity to develop greater levels of general self-efficacy and nursing self-efficacy; however, there needs to be a greater emphasis on developing the elements of resilience, optimism, efficacy, and hope, especially among first-year students, and prior to entering the workplace when it is most adaptable. Currently, it has been argued this remains absent or ineffectual in the undergraduate and workplace spheres [16]. As such, regardless of the workplace or learning situation, the development of self-efficacy is achieved through the four main sources of information, as proposed by Bandura [8]. These include (1) performance accomplishments or personal mastery, (2) vicarious (or visualized) experiences of others accomplishments, (3) verbal (social) persuasion, and (4) emotional (psychological) arousal leading to aversive or motivating behaviors [8]. 

More specifically, Bandura [8], in his seminal work, outlines how self-efficacy is developed through personal mastery or performance accomplishments, which is achieved through repeated successes and intermittent failures, inadequacies where improvements and mastery are developed through self-motivated persistence and effort. For nursing students, this may be about reflecting on practice, identifying areas of weakness, and making the required changes to improve practice. It is these personal accomplishments and achievements that develop self-efficacy that are transferable to other similar and even divergent situations. 

Similarly, the notion of vicarious or visualized experiences of others’ accomplishments with clear outcomes can also have analogous impact on self-efficacy. Seeing others perform activities or successfully achieving goals, through social comparison, engenders expectations among observers that ‘If they can do it, so can I’. Seeing others’ successful performance and outcomes increases the observer’s sense of self-efficacy. This modelling is often less powerful to developing self-efficacy, however, is dependent on the context or situation in which it is used [8]. For example, a nursing student may gain greater self-efficacy from vicarious experiences that are observed in the classroom or clinical settings prior to their own actual nursing performance [4,8]. 

In addition to personal mastery, verbal persuasion, a more often used approach, can increase self-efficacy through telling someone what can be achieved, and persuasion of the outcome. In this sense, nursing students may gain greater self-efficacy through verbal persuasion from each other, preceptors, and academics. However, this approach is less authentic due to its inability to provide experiential outcomes, and the capacity of the individual to dissuade or justify to themselves [8].

Lastly, emotional arousal can lead to assertive or motivating behaviors, but can also have a negative effect on self-efficacy and coping. It is this capacity to rely on the emotional state, anxiety, fear, or stress that can be crippling. However, these emotional states, when acknowledged and appraised, can be informative and motivate what the action may be. Among nursing students, this may be about identifying when anxieties, fears, and stressors occur in practice. As such, emotional states can be used as a source of information to identify coping strategies when they occur, or to be used in conjunction with modeling. This may then abate avoidance behaviors, the strongest predictor of burnout among nursing students, which emerges from emotional arousal [4,8,17]. 

### 4.2. Limitations

Overall, the university has campuses in rural, regional, and peri-urban locations with a large student cohort from rural settings. This may limit the ability to generalize the findings as many universities are located in more metropolitan centers. In addition, student respondents of the survey may not be representative of the whole student cohort given the low response rate, with only 10.2% of the student cohort participating in the survey in full. The low response rate may be due to the survey being administered in the mid-semester break. To increase response rate without increasing coercion, the survey may be more suited to be administered at the beginning of the year.

## 5. Conclusion

Overall, it is evident from the study that a greater emphasis or a more tailored effort is required to develop general self-efficacy, and particularly nursing self-efficacy, among those students who are first-year students, who may not have any previous nursing experience, and who demonstrate lower levels of hope, resilience, and optimism. Addressing self-efficacy after a student has graduated remains problematic, as self-efficacy is more malleable during the education period of training and becomes more resistant to change after employment has occurred [8]. As such, self-efficacy within nursing must commence early in the first year, and its development should be actively increased through performance accomplishments, vicarious experiences of others, verbal and social persuasion, and emotional arousal, which are considered essential practices in nurse education [8]. 

The findings suggest that greater efforts are needed to further develop occupationally specific self-efficacy. Students’ self-efficacy must be actively nurtured and developed so that they can cope better as they enter a workforce where there is an increasing complexity of patient care, new technologies to learn, increased overtime, ever-burgeoning staffing demands, and a decreasing support for graduate nurses. In addition, by assisting all students to develop certain elements of psychological capital such as hope and optimism, nursing students may develop a greater capacity to bounce back and overcome the challenges that they may encounter in their undergraduate academic training, and to reduce attrition as they prepare to enter the workplace. As such, we are suggesting to education providers that undergraduate nursing training must move beyond the theoretical tenets of nursing and skills acquisition to embrace higher education that is centered on developing occupational self-efficacy and psychological capital. In doing so, students are enabled to enter the nursing workforce equipped with the foundational “inner” attributes for a long and satisfying career in nursing.

## Figures and Tables

**Table 1:** Participant demographics.

Demographic Information	Frequency	Percentage (%)
Gender (n = 286)		
− Female	261	91.2
− Male	24	8.3
− Other	1	0.5
Year of program (n = 284)		
− First year	85	30.0
− Second year	100	35.2
− Third year	99	34.8
Age (years) (n = 269)		
− Under 25	92	34.2
− 25–45 years	149	55.4
− Over 45 years	28	10.4
Where grew up (n = 274)		
− Metropolitan area	89	32.5
− Regional area	66	24.0
− Rural (property or farm or rural town)	119	43.5
Employment status (n = 280)		
− Full-time	30	9.0
− Part-time	110	32.7
− Casual (Temporary)	88	26.2
− Unemployed	52	15.5
Current after tax income a week (n = 277)		
Less than $400	123	44.4
− $400–$799	87	31.4
− $800–$1499	38	13.7
− $1500–$3000	4	1.4
− Do not wish to answer	26	9.4
Enrolled Nurse: (n = 284)		
− Yes	60	21.1
− No	224	78.9

**Table 2:** Comparison of mean scale items between groups using t-test or ANOVA.

Scale Item	Factor	Group	Mean (SD)	Test (df) Statistic	*p*
GSE-10–NSE-8	-	-	31.89(3.89)	t(207) = 7.314	0.001 ***
			23.91(3.01)		
GSE	Where students grew up	− Metropolitan	32.85(3.49)	F(2, 198) = 2.883	0.050 *
		− Rural	31.32(4.34)		
NSE	Where students grew up	− Metropolitan	24.56(2.64)	F(2, 198) = 4.284	0.014 *
		− Rural	23.15(3.34)		
Efficacy	Age	− Under 25	4.11(1.14)	F(2, 203) = 4.119	0.015 *
		− 25–45	4.59(0.99)		
Optimism	Where students grew up	− Metropolitan	5.04(0.75)	F(2, 210) = 4.530	0.016 *
		− Regional	4.53(0.83)		
Optimism	Where students grew up	− Metropolitan	5.05(0.75)	F(2, 210) = 4.666	0.022 *
		− Rural	4.66(0.98)		
Hope	Employment	− Employed	4.49(1.01)	t(217) = 8.960	0.003 **
		− Unemployed	4.70(0.89)		
Resilience	Employment	− Employed	4.49(0.99)	t(217) = 7.249	0.008 **
		− Unemployed	4.85(0.98)		

*p* < 0.05 *, *p* < 0.01 **, *p* < 0.001 ***.

**Table 3:** Comparison of mean scale items between years using ANOVA.

Scale Item	Group 1	Mean (SD)	Group 2	Mean (SD)	Test (df) Statistic	*p*
NSE	First year	3.06(0.55)	Second year	3.36(0.45)	F(2, 198) = 9.815	0.001 ***
			Third year	3.39(0.51)		
Efficacy	First year	4.32(1.08)	Third year	4.80(0.96)	F(2, 203) = 4.609	0.008 **
Hope	First year	4.63(0.87)	Third year	5.04(0.83)	F(2, 215) = 5.095	0.007 **
Optimism	First year	4.68(1.04)	Second year	5.03(1.01)	F(2, 210) = 5.953	0.022 *
			Third year	5.15(0.80)		0.004 *

*p* < 0.05 *, *p* < 0.01 **, *p* < 0.001 ***.

**Table 4:** Final models of general self-efficacy and nursing self-efficacy using multiple regression.

Model	Variable	Mean (SD)	Combined R^2^	β	t	*p*
GSE model	Age	32.54(10.14)	0.422	0.186	2.819	0.005 **
	Efficacy	4.42(1.02)		0.167	2.277	0.024 *
	Hope	4.63(0.89)		0.318	4.031	0.001 ***
	Optimism	4.74(0.95)		0.269	3.843	0.001 ***
	Income level	-		−0.168	−2.570	0.011 ***
NSE model	Hope	4.63(0.89)	0.165	0.256	3.561	0.001 ***
	Optimism	4.74(0.95)		0.223	2.796	0.006 **
	Enrolled nurse	-		0.160	2.265	0.025 *
	Income level	-		0.156	2.217	0.028 *

*p* < 0.05 *, *p* < 0.01 **, *p* < 0.001 ***.

**Table 5:** Correlation between general and nursing self-efficacy and psychological capital.

Variable (n = 208)		GSE	NSE
NSE	Pearson (r)	0.458 *	-
	Sig. (2-tailed)	0.005	-
	n	208	
Efficacy	Pearson (r)	0.486 **	0.256 **
	Sig. (2-tailed)	0.005	0.005
	n	208	208
Hope	Pearson (r)	0.520 **	0.249 **
	Sig. (2-tailed)	0.005	0.005
	n	208	208
Resilience	Pearson (r)	0.418 **	0.135
	Sig. (2-tailed)	0.005	0.250
	n	208	208
Optimism	Pearson (r)	0.516 **	0.284 **
	Sig. (2-tailed)	0.005	0.005
	n	208	208

*p* < 0.05 *, *p* < 0.01 **, *p* < 0.001 ***.

**Table 6:** Correlation between general and nursing self-efficacy and psychological capital among age groups.

Variable		GSE	NSE	GSE	NSE	GSE	NSE
		Under 25 (n = 61)		25–45 (n = 112)		Over 45 (n = 23)	
NSE	Pearson (r)	0.615 **	-	0.364 **	-	0.421 **	-
	Sig. (2-tailed)	0.005	-	0.005	-	0.005	-
	n	61		112		23	
Efficacy	Pearson (r)	0.600 **	0.431 **	0.359 **	0.151	0.408	0.106
	Sig. (2-tailed)	0.005	0.005	0.005	0.447	0.242	0.993
	n	59	61	112	112	23	23
Hope	Pearson (r)	0.536 **	0.457 **	0.548 **	0.325 **	0.521	−0.072
	Sig. (2-tailed)	0.005	0.005	0.005	0.005	0.053	0.998
	n	61	61	112	112	23	23
Resilience	Pearson (r)	0.573 **	0.344 **	0.392 **	0.171	0.379	−0.074
	Sig. (2-tailed)	0.005	0.034	0.005	0.308	0.322	0.998
	n	61	61	112	112	23	23
Optimism	Pearson (r)	0.464 **	0.315	0.559 **	0.329 **	0.373	0.038
	Sig. (2-tailed)	0.005	0.063	0.005	0.005	0.337	0.998
	n	61	61	112	112	23	23

*p* < 0.05 *, *p* < 0.01 **, *p* < 0.001 ***.

**Table 7:** Correlation between general and nursing self-efficacy and psychological capital according to where students grew up.

Variable		GSE	NSE	GSE	NSE	GSE	NSE
		Metropolitan (n = 62)		Regional (n = 46)		Rural (n = 91)	
NSE	Pearson (r)	0.325 *	-	0.466 **	-	0.456 **	-
	Sig. (2-tailed)	0.049	-	0.005	-	0.005	-
	N	62		46		91	
Efficacy	Pearson (r)	0.334 *	0.265	0.257	−0.147	0.561 **	0.314 **
	Sig. (2-tailed)	0.039	0.171	0.358	0.863	0.005	0.009
	n	62	62	46	46	91	91
Hope	Pearson (r)	0.390 **	0.405 **	0.478 **	0.113	0.603 **	0.313 **
	Sig. (2-tailed)	0.009	0.005	0.005	0.951	0.005	0.009
	n	62	62	46	46	91	91
Resilience	Pearson (r)	0.399 **	0.094	0.323	0.141	0.502 **	0.218
	Sig. (2-tailed)	0.005	0.957	0.136	0.883	0.005	0.176
	n	62	62	46	46	91	91
Optimism	Pearson (r)	0.408 **	0.169	0.401 **	0.266	0.577 **	0.353 **
	Sig. (2-tailed)	0.005	0.646	0.029	0.319	0.005	0.005
	n	62	62	46	46	91	91

*p* < 0.05 *, *p* < 0.01 **, *p* < 0.001 ***.

**Table 8:** Correlation between general and nursing self-efficacy and psychological capital among students who are enrolled nurses and those who are not.

Variable		GSE	NSE	GSE	NSE
		Enrolled Nurses (N = 44)		Not Enrolled Nurses (n = 164)	
NSE	Pearson (r)	0.528 **	-	0.455 **	-
	Sig. (2-tailed)	0.005	-	0.005	-
	n	44		164	
Efficacy	Pearson (r)	0.297	0.098	0.483 **	0.276 **
	Sig. (2-tailed)	0.226	0.976	0.005	0.005
	n	44	44	164	164
Hope	Pearson (r)	0.593 **	0.308	0.500 **	0.292 **
	Sig. (2-tailed)	0.005	0.193	0.005	0.005
	n	44	44	164	164
Resilience	Pearson (r)	0.593 **	0.173	0.384 **	0.160
	Sig. (2-tailed)	0.005	0.781	0.005	0.188
	n	44	44	164	164
Optimism	Pearson (r)	0.622 **	0.512 **	0.488 *	0.280 *
	Sig. (2-tailed)	0.005	0.005	0.005	0.005
	n	44	44	164	164

*p* < 0.05 *, *p* < 0.01 **, *p* < 0.001 ***.

## References

[1] Mills J., Woods C., Harrison H., Chamberlain-Salaun J., Spencer B. (2017). Retention of early career registered nurses: The influence of self-concept, practice environment and resilience in the first five years post-graduation. J. Res. Nurs..

[2] Yılmaz E.B. (2017). Resilience as a strategy for struggling against challenges related to the nursing profession. Chin. Nurs. Res..

[3] Guarnaccia C., Scrima F., Civilleri A., Salerno L. (2018). The role of occupational self-efficacy in mediating the effect of job insecurity on work engagement, satisfaction and general health. Curr. Psychol..

[4] Luthans F., Luthans K.W., Luthans B.C. (2004). Positive psychological capital: Beyond human and social capital. Bus. Horiz..

[5] Bandura A., Locke E.A. (2003). Negative self-efficacy and goal effects revisited. J. Appl. Psychol..

[6] McLaughlin K., Moutray M., Muldoon O.T. (2008). The role of personality and self-efficacy in the selection and retention of successful nursing students: A longitudinal study. J. Adv. Nurs..

[7] Lorenz T., Beer C., Pütz J., Heinitz K. (2016). Measuring psychological capital: Construction and validation of the compound PsyCap scale (CPC-12). PLoS ONE.

[8] Bandura A. (1977). Self-efficacy: Toward a unifying theory of behavioral change. Psychol. Rev..

[9] Ward T., Durrant R. (2011). Evolutionary psychology and the rehabilitation of offenders: Constraints and consequences. Aggress. Violent Behav..

[10] DeWitz S.J., Woolsey M.L., Walsh W.B. (2009). College student retention: An exploration of the relationship between self-efficacy beliefs and purpose in life among college students. J. Coll. Stud. Dev..

[11] Schyns B. (2004). The influence of occupational self-efficacy on the relationship of leadership behavior and preparedness for occupational change. J. Career Dev..

[12] Pisanti R., Lombardo C., Lucidi F., Lazzari D., Bertini M. (2008). Development and validation of a brief occupational coping self-efficacy questionnaire for nurses. J. Adv. Nurs..

[13] Boamah S., Laschinger H. (2015). Engaging new nurses: The role of psychological capital and workplace empowerment. J. Res. Nurs..

[14] Avey J.B., Luthans F., Jensen S.M. (2009). Psychological capital: A positive resource for combating employee stress and turnover. Hum. Resour. Manag..

[15] Luthans F., Avolio B.J., Avey J.B., Norman S.M. (2007). Positive psychological capital: Measurement and relationship with performance and satisfaction. Pers. Psychol..

[16] Kennedy E. (2013). The Nursing Competence Self-Efficacy Scale: An Instrument Development and Psychometric Assessment Study.

[17] Gibbons C. (2010). Stress, coping and burn-out in nursing students. Int. J. Nurs. Stud..

[18] Townsend L., Scanlan J.M. (2011). Self-efficacy related to student nurses in the clinical setting: A concept analysis. Int. J. Nurs. Educ. Scholarsh..

[19] Cheraghi F., Hassani P., Yaghmaei F., Alavi-Majed H. (2009). Developing a valid and reliable self-efficacy in clinical performance scale. Int. Nurs. Rev..

[20] Terry D., Peck B., Smith A., Stevenson T., Baker E. (2019). Is nursing student personality important for considering a rural career?. J. Health Organ. Manag..

[21] Schwarzer R., Jerusalem M. (2010). The general self-efficacy scale (GSE). Anxiety Stress Coping.

[22] Schyns B., Von Collani G. (2002). A new occupational self-efficacy scale and its relation to personality constructs and organizational variables. Eur. J. Work Organ. Psychol..

[23] Luthans F., Avolio B.J., Avey J.B. (2007). Psychological Capital (PsyCap) Questionnaire (PCQ).

[24] Palant J. (2013). SPSS Survival Manual: A Step by Step Guide to Data Analysis Using IBM SPSS.

[25] Isaac V., Walters L., McLachlan C.S. (2015). Association between self-efficacy, career interest and rural career intent in Australian medical students with rural clinical school experience. BMJ Open.

[26] Casapulla S.L. (2017). Self-efficacy of Osteopathic Medical Students in a Rural-Urban Underserved Pathway Program. J. Am. Osteopath. Assoc..

